# Production and Reutilization of Fluorescent Dissolved Organic Matter by a Marine Bacterial Strain, *Alteromonas macleodii*

**DOI:** 10.3389/fmicb.2017.00507

**Published:** 2017-03-28

**Authors:** Shuji Goto, Yuya Tada, Koji Suzuki, Youhei Yamashita

**Affiliations:** ^1^Graduate School of Environmental Science, Hokkaido UniversitySapporo, Japan; ^2^Faculty of Environmental Earth Science, Hokkaido UniversitySapporo, Japan; ^3^Project Team for Research and Development of Next-generation Technology for Ocean Resources Exploration, Japan Agency for Marine-Earth Science and TechnologyYokosuka, Japan

**Keywords:** microbial carbon pump, recalcitrant DOM, fluorescent DOM, *Alteromonas macleodii*, growth phase, EEMs

## Abstract

The recalcitrant fraction of marine dissolved organic matter (DOM) plays an important role in carbon storage on the earth’s surface. Bacterial production of recalcitrant DOM (RDOM) has been proposed as a carbon sequestration process. It is still unclear whether bacterial physiology can affect RDOM production. In this study, we conducted a batch culture using the marine bacterial isolate *Alteromonas macleodii*, a ubiquitous gammaproteobacterium, to evaluate the linkage between bacterial growth and DOM production. Glucose (1 mmol C L^-1^) was used as the sole carbon source, and the bacterial number, the DOM concentration in terms of carbon, and the excitation–emission matrices (EEMs) of DOM were monitored during the 168-h incubation. The incubation period was partitioned into the exponential growth (0–24 h) and stationary phases (24–168 h) based on the growth curve. Although the DOM concentration decreased during the exponential growth phase due to glucose consumption, it remained stable during the stationary phase, corresponding to approximately 4% of the initial glucose in terms of carbon. Distinct fluorophores were not evident in the EEMs at the beginning of the incubation, but DOM produced by the strain exhibited five fluorescent peaks during exponential growth. Two fluorescent peaks were similar to protein-like fluorophores, while the others could be categorized as humic-like fluorophores. All fluorophores increased during the exponential growth phase. The tryptophan-like fluorophore decreased during the stationary phase, suggesting that the strain reused the large exopolymer. The tyrosine-like fluorophore seemed to be stable during the stationary phase, implying that the production of tyrosine-containing small peptides through the degradation of exopolymers was correlated with the reutilization of the tyrosine-like fluorophore. Two humic-like fluorophores that showed emission maxima at the longer wavelength (525 nm) increased during the stationary phase, while the other humic-like fluorophore, which had a shorter emission wavelength (400 nm) and was categorized as recalcitrant, was stable. These humic-like fluorophore behaviors during incubation indicated that the composition of bacterial humic-like fluorophores, which were unavailable to the strain, differed between growth phases. Our results suggest that bacterial physiology can affect RDOM production and accumulation in the ocean interior.

## Introduction

Marine dissolved organic matter (DOM) is one of the largest reduced organic carbon pools on the earth’s surface, indicating that it plays as important role of the total carbon pool in the ocean (Hedges, 1992; Hansell and Carlson, 1998; Hansell et al., 2009). The average residence time of bulk DOM in the ocean has been estimated at approximately 2000–6000 years by ^14^C dating analysis (Bauer et al., 1992; Druffel et al., 1992; Beaupré,, 2015). Observations of the global distribution of dissolved organic carbon (DOC) concentration with a coupled physical/biogeochemical model also showed that a major fraction of marine DOM is recalcitrant to microbial degradation with a time scale of more than a century (Hansell et al., 2009). Some constituents of marine DOM, e.g., humic-like fluorescent DOM (FDOM) (Yamashita and Tanoue, 2008; Catalá et al., 2015), carboxyl-rich aliphatic materials (Hertkorn et al., 2006), and polyaromatic compounds (Dittmar and Paeng, 2009), have been considered to be recalcitrant DOM (RDOM). However, the chemical characteristics of RDOM have not been fully clarified. Such molecularly uncharacterizable features of RDOM preclude a comprehensive understanding of the source and production mechanism of marine RDOM.

The microbial carbon pump (MCP) has recently been proposed as a carbon sequestration process driven by bacterial RDOM generation (Jiao et al., 2010). The MCP concept was derived from the results of microbial incubation studies (Ogawa et al., 2001; Kramer and Herndl, 2004; Kawasaki and Benner, 2006; Lønborg et al., 2009; Shimotori et al., 2009; Koch et al., 2014). These studies observed that DOM, which was distinct from labile substrates (such as glucose and glutamate) for microbes, was present in 20-day to 2-year incubations of microbial communities obtained from seawater. The residual DOM has been considered to be microbially derived DOM, which could not be utilized by heterotrophic bacteria during incubation. Ultrahigh resolution Fourier transform ion cyclotron resonance mass spectrometry (FT-ICR-MS) has recently been applied to determine the molecular composition of DOM obtained from in vitro incubations of marine microbial community. The results showed that experimentally obtained microbial DOM was similar to marine RDOM in terms of the molecular composition (Koch et al., 2014; Lechtenfeld et al., 2015), although it has also been reported that most microbial DOM is distinct from marine RDOM (Osterholz et al., 2015).

The products of MCP have also been traced using fluorescence techniques, e.g., excitation–emission matrices (EEMs), during in vitro incubation of marine microbial communities with various substrates, e.g., simple substrate, such as glucose (Kramer and Herndl, 2004; Lønborg et al., 2009; Shimotori et al., 2009), marine DOM (Jørgensen et al., 2014), and humic substances (Aparicio et al., 2015). These studies found that humic-like fluorophores were produced by marine microbes, and microbial humic-like fluorophores were usually not degraded during incubation. Shimotori et al. (2009) conducted a 90-day incubation experiment using a coastal microbial community with glucose as substrate, and reported that the coastal bacterial community generated a humic-like fluorophore that was similar to marine RDOM in terms of fluorescence characteristics, molecular size, and photo-degradability. Recent studies also suggest that production rates of humic-like fluorophore by marine microbial communities depended on the quality of substrates (Jørgensen et al., 2014; Aparicio et al., 2015). Such substrate dependency of humic-like fluorophore production by microbial communities is possibly due to changes in bacterial physiology and/or responding species with different substrates.

It has been suggested that the microbial RDOM is produced through bacterial exudation (Jiao et al., 2010, 2014), indicating a potential relationship between bacterial physiology and RDOM production. However, the relationship of RDOM production with bacterial physiology has scarcely been discussed because incubation experiments for evaluating MCP have usually been conducted with natural microbial communities, which include a wide variety of microbes with various physiologies. The incubation of a bacterial isolate could allow the evaluation of bacterial physiological states, as well as bacterial growth phase; yet, only few studies have been conducted on this topic. The FT-ICR-MS-based exometabolomics analysis of *Pseudovibrio* sp. incubation showed that the composition of bacterial DOM was affected by the growth phase (Romano et al., 2014). In contrast, Gruber et al. (2006) suggested that *Pseudomonas chlororaphis* produced persistent DOM, mainly during the exponential growth phase. On the other hand, Eichinger et al. (2009) reported that DOC accumulated during the stationary phase in the incubation of *Alteromonas infernus*. At present, only a few studies have assessed the microbial production of RDOM using marine bacterial isolates, and therefore, knowledge of the relationship between RDOM production and bacterial physiology is still limited, even though it is possibly one of the key parameters shaping the size and composition of the marine DOM pool.

The objective of this study was to investigate the relationship between physiology in terms of growth phase and RDOM production through in vitro incubation of a model marine bacterial isolate with glucose as the sole substrate. *Alteromonas macleodii*, a ubiquitous gammaproteobacterium from the surface to the deep layer of tropical and temperate oceans (López-Pérez et al., 2012), was used as a model marine bacterial isolate in this study. *Alteromonas* was reported to grow predominantly during diatom blooms in the western North Pacific Ocean (Tada et al., 2011). An *Alteromonas* sp., designated strain AltSIO, was isolated by the Scripps Institution of Oceanography from coastal seawater and found to share ∼99% 16S ribosomal DNA sequence similarity with *A. macleodii* (Pedler et al., 2014; Pedler Sherwood et al., 2015). AltSIO alone consumed the entire pool of labile DOC, defined as the quantity consumed by coastal microbial assemblages within 5 days (Pedler et al., 2014), and has the capacity to significantly alter marine DOM composition (Pedler Sherwood et al., 2015). These studies suggested that *A. macleodii* also might contribute to high consumption and alteration of labile DOM in the ocean’s surface. In this study, the humic-like fluorophores determined by EEMs were used to evaluate bacterial RDOM, while protein-like fluorophores in EEMs were used to monitor the reuse of the bacterial exopolymer.

## Materials and Methods

### Marine Bacterial Isolate Model

The strain *A. macleodii* ATCC 27126 was obtained from the Japan Collection of Microorganisms, RIKEN Bio Resource Center (Tsukuba, Ibaraki, Japan), and was used as a model marine bacterial isolate.

Prior to the present experimental setup, 100 μL frozen stock of *A. macleodii* was inoculated onto 100 mL artificial seawater-based Aquil medium (Price et al., 1989) supplemented with glucose (1 mmol C L^-1^) at 25°C for 24 h. Then, the medium was transferred onto new Aquil medium and incubated under the same conditions as the pre-culture medium. After 24 h of incubation, the culture was used as inoculum for the present experiment.

### Experimental Setup

*Alteromonas macleodii* was cultivated in modified organic carbon-free Aquil medium (Price et al., 1989) (**Table 1**). Anhydrous salts used for artificial seawater (NaCl, Na_2_SO_4_, KCl, KBr) were combusted at 450°C for 4 h. NaH_2_PO_4_⋅H_2_O (10 μmol P L^-1^) and NaNO_3_ (161 μmol N L^-1^) were added as major nutrients. The media for control and experimental treatments were prepared without and with glucose (1 mmol C L^-1^) in each triplicate, respectively. The *A. macleodii* inoculum was added to the medium of each treatment condition at a 1:1000 dilution. Then, each treatment condition with *A. macleodii* inoculum was dispensed into acid-washed (1 M HCl) 250 mL polyethylene terephthalate bottles. The bottle was filled with 100 mL of culture medium, and thus, gas phase was maintained in the bottles to keep oxygen at a high enough level to catabolize all of the added glucose. Incubations of the experimental and control treatments were conducted in the dark and in an incubator (CN-40, Mitsubishi-Engineering Co.) in which the temperature was maintained at 25°C. To determine the bacterial abundance, DOC concentration and DOM optical property, triplicate bottles were sampled at 0, 6, 12, 18 24, 72, 120, and 168 h in the experimental treatment and at 0 and 168 hours in the control treatment.

**Table 1:** Composition of modified Aquil medium.

	Compound	Final concentration (mol L^-1^)
Salts	NaCl	4.20 × 10^-1^
	Na_2_SO_4_	2.88 × 10^-2^
	KCl	9.39 × 10^-3^
	NaHCO_3_	2.38 × 10^-3^
	KBr	8.40 × 10^-4^
	H_2_BO_3_	4.85 × 10^-5^
	NaF	7.15 × 10^-5^
	MgCl_2_⋅6H_2_O	5.46 × 10^-2^
	CaCl_2_⋅2H_2_O	1.05 × 10^-2^
	SrCI_2_⋅6H_2_O	6.38 × 10^-5^
Trace metal	FeCl_2_⋅6H_2_O	1.00 × 10^-6^
	ZnSO_4_⋅7H_2_O	7.97 × 10^-8^
	MnCl_2_⋅4H_2_O	1.21 × 10^-7^
	CoCI_2_⋅6H_2_O	5.03 × 10^-8^
	CuSO_4_⋅5H_2_O	1.96 × 10^-8^
	Na_2_MoO_4_⋅2H_2_O	1.00 × 10^-7^
	Na_2_SeO_3_	1.00 × 10^-8^
Major nutrients	NaHPO_4_⋅H_2_O	1.00 × 10^-5^
	NaNO_3_	1.61 × 10^-4^

### Bacterial Abundance

Bacterial cell density was measured with an EPICS flow cytometer (XL ADC system, Beckman Coulter) equipped with a 15 mW air-cooled laser exciting at 488 nm, according to the protocol of Tada and Suzuki (2016). Samples were fixed in paraformaldehyde [2% (vol/vol) final concentration] and preserved at –25°C. Just before analysis, samples were stained with SYBR Gold (SYBR Gold Nucleic Acid Gel Stain, Life technologies) at a final concentration of 10^-4^ commercial stock solution for at least 15 min. To calculate the flow rate, 2 μm fluorescent beads (Fluoresbrite YG Carboxylate Microspheres 2.00 μm, Polysciences, Inc.) were added to the flow samples. We used a low flow rate mode and analyzed the samples until twenty thousand particles were counted or the measurement time reached 5 minutes. The measured values were corrected by blank value subtraction; the blank was measured with artificial seawater filtered with 0.2-μm pore size cellulose membrane filters (DISMIC-25AS 0.20 μm, ADVANTEC).

### DOC Concentrations and DOM Optical Properties

Samples for DOM analyses were filtered through pre-combusted (450°C, 3 h) glass fiber filters with a nominal pore size of 0.3 μm (GF75, Whatman) under gentle vacuum (<0.02 MPa) at each incubation time to remove particles, including *A. macleodii*. The filtrate was collected into a pre-combusted (450°C, 3 h) glass vial with teflon-lined cap and was preserved at –25°C until analysis.

The DOC concentration was determined by high-temperature catalytic oxidation with a total organic carbon analyzer (TOC-V CSH, Shimadzu). The DOC concentrations were calculated using the standard curve of potassium hydrogen phthalate solution, which was determined daily. The accuracy and consistency of the measured DOC concentrations were checked by a deep seawater reference sample (Hansell Laboratory, University of Miami), which was assessed daily.

Excitation–emission matrix was measured using a fluorometer (FluoroMax-4, Horiba) according to the procedure of Tanaka et al. (2014). Samples were allowed to reach near room temperature before the EEM measurements were undertaken. Forty-one emission scans from 290 to 600 nm taken at 2-nm intervals were acquired for the excitation wavelengths between 250 and 450 nm at 5-nm intervals. The bandpass was set to 5 nm for both excitation and emission. The fluorescence spectra were scanned with a 0.25 s integration time and acquired in the S/R mode. Several post-acquisition steps were involved in the correction of the fluorescence spectra, including instrumental bias correction and corrections of inner filter effect using absorbance. Following this, the EEMs of Milli-Q water were subtracted from those of the samples, and fluorescence intensities in EEMs were converted to Raman Units (RU) with the peak areas of Raman scatter at 350 nm excitation (Lawaetz and Stedmon, 2009). RU can be converted to quinine sulfate units (QSU) by using the equation QSU = RU/0.0767 (Lawaetz and Stedmon, 2009). The absorbance spectrum of each sample for correction of inner filter effect was measured with a Shimadzu UV-1800 spectrophotometer in a 1-cm quartz cuvette according to Yamashita et al. (2013). Because nitrate has relatively high absorbance in the UV-B region (Catalá et al., 2016) at high concentrations (e.g., 161 μmol N L^-1^ in the medium), investigations of changes in the absorption spectrum associated with *A. macleodii* incubation were abandoned. The contour of EEMs was plotted by R (version 3.2.3) (R Development Core Team, 2015).

## Results

### Growth of *A. macleodii* and Change in DOC Concentration

**FIGURE 1:**
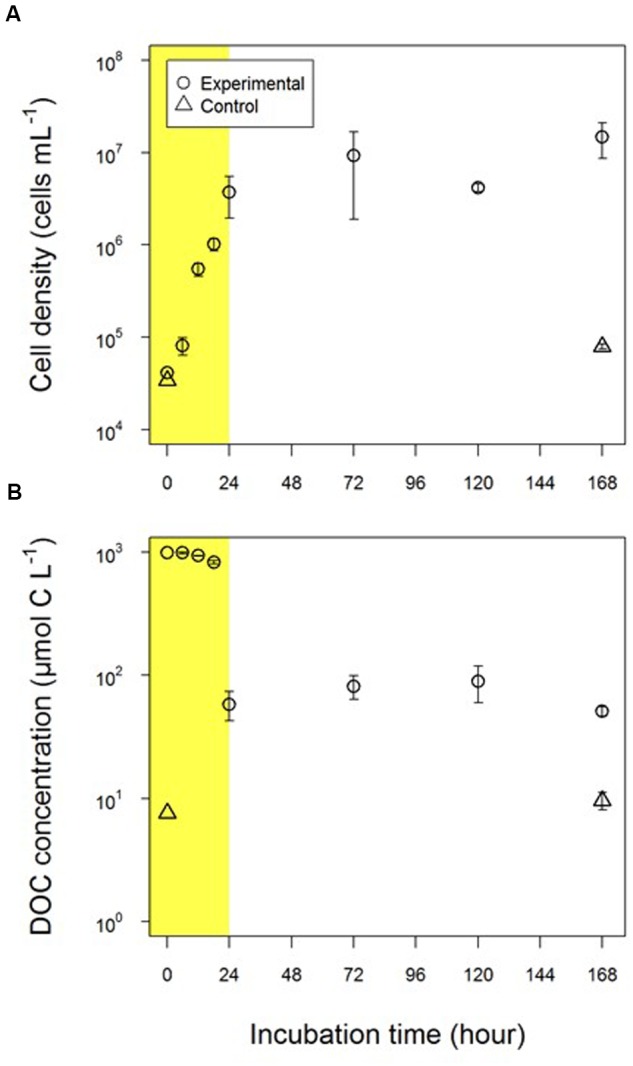
**Changes in (A)** the abundance of *Alteromonas macleodii* (*A. macleodii*) and **(B)** the concentration of dissolved organic carbon (DOC). Circles and triangles represent the experimental and control treatments, respectively. Yellow shading indicates the exponential growth phase.

The abundance of *A. macleodii* increased exponentially during the first 24 h in the experimental treatment (**Figure 1A**). The cell density was 4.1 × 10^4^ cells mL^-1^ at the initiation of incubation (*t* = 0) and increased up to 3.7 ± 1.8 × 10^6^ cells mL^-1^ after 24 h. The cell density then remained stable on a logarithmic scale until the end of the experiment (**Figure 1A**). Therefore, the periods of 0–24 h and 24–168 h were defined as the exponential growth and stationary phases, respectively. The average specific growth rate was 0.19 ± 0.10 h^-1^ during the exponential growth phase. In the control treatment, cell density increased approximately twofold during the incubation (from 3.4 × 10^4^ cells mL^-1^ at the initiation of the incubation to 8.0 ± 0.5 × 10^4^ cells mL^-1^ at the end of the incubation), indicating that bacterial growth caused by the medium substrate and carbon storage in the cells of the inoculum was much lower than that caused by glucose in the experimental treatment.

The DOC concentration gradually decreased during the early part of the exponential growth phase (first 18-h) from 995 μmol C L^-1^ to 830 ± 28 μmol C L^-1^ (**Figure 1B**) and then drastically decreased to 58 ± 15 μmol C L^-1^ in the latter part of the exponential growth phase (18–24 h). There was a larger relative decrease in DOC concentration normalized by bacterial cell density between 18 and 24 h (0.29 mmol C cell^-1^) than between 0 and 18 h (0.17 mmol C cell^-1^), thus suggesting a substantial increase in *A. macleodii* cell volume between 18 and 24 h. The DOC concentration was almost constant (from 51 ± 4 μmol C L^-1^ to 89 ± 29 μmol C L^-1^) over the stationary phase and was 51 ± 4 μmol C L^-1^ at the end of the experimental incubations (168 h).

The DOC concentrations in the control treatment were 1–2 orders of magnitude lower than those in the experimental treatment and were 7.6 μmol C L^-1^ and 9.6 ± 1.5 μmol C L^-1^ at the beginning and end of the incubation, respectively. The DOC concentrations detected in the control experiment were possibly derived from the medium and bacterial inoculum.

### FDOM Derived from *A. macleodii*

**FIGURE 2:**
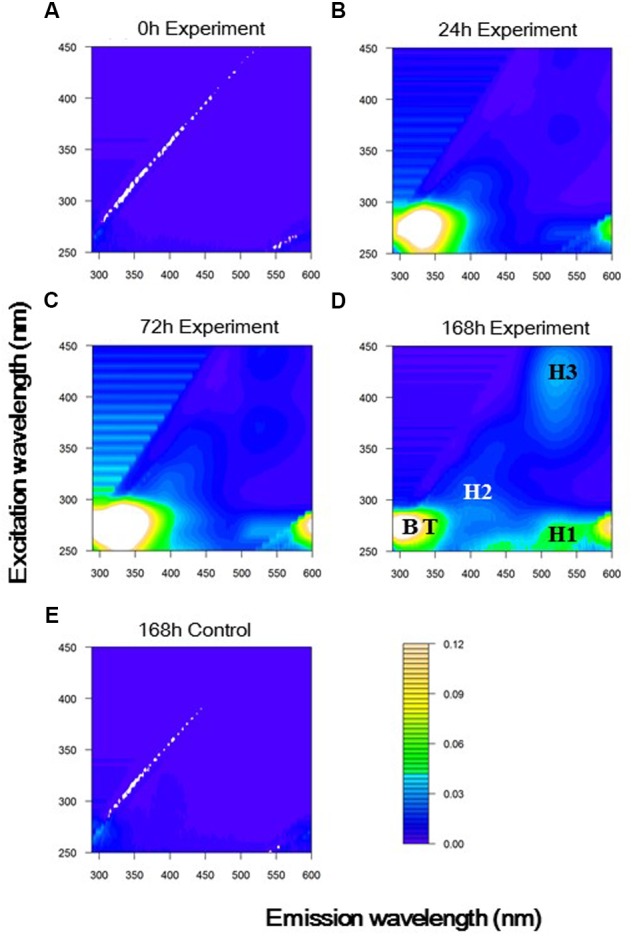
**Excitation-emission matrices (EEMs) based on the average of triplicate samples at (A)** 0, **(B)** 24, **(C)** 72, and **(D)** 168 h in the experimental treatment, and at **(E)** 168 h in the control treatment. Peaks generated by *A. macleodii* were indicated with abbreviations: Tyrosine-like (B, Ex/Em = 275/300 nm); Tryptophan-like (T, Ex/Em = 275/330 nm); Humic-like 1 (H1, Ex/Em = 270/520 nm); Humic-like 2 (H2, Ex/Em = 315/400 nm); Humic-like 3 (H3, Ex/Em = 425/520 nm).

Fluorescent peaks found in EEMs at the 24-h time point and the end of the experimental treatment are shown in **Figures 2B,D**, respectively. Because EEMs at the beginning of the experimental treatment (**Figure 2A**) and at the end of the control treatment (**Figure 2E**) did not show distinct fluorescent peaks, it can be concluded that fluorescent peaks found for the experimental treatment were derived from *A. macleodii* and not from the incubation medium or contamination during incubation.

Five fluorescent peaks were defined from EEMs obtained at the end of the experimental treatment (**Figure 2D** and **Table 2**). Two peaks were characterized as protein-like fluorescent peaks similar to the aromatic amino acids tyrosine and tryptophan (Coble, 1996; Mayer et al., 1999). From EEMs observed during the experimental treatment (**Figures 2B–D**), the excitation and emission wavelength (Ex/Em) of tyrosine-like and tryptophan-like peaks were defined to be 275/300 nm and 275/330 nm, respectively. Three peaks could be categorized to humic-like fluorophores. Two of them had emission maximum longer than 500 nm (H1: Ex/Em = 270/520 nm, H3: Ex/Em = 425/520 nm) and have usually been defined to be terrestrial humic-like FDOM in coastal environments (Stedmon and Markager, 2005). The combined fluorescence characteristics of H1 and H3 were similar to that found in the most abundant component in the humic acid fraction extracted from sediments/soils (He et al., 2006; Santín et al., 2009). The other humic-like fluorophore (H2: Ex/Em = 315/400 nm), which had a shorter emission wavelength than H1 and H3, was similar to traditionally defined marine/microbial humic-like FDOM (Coble, 1996, 2007) and was also similar to a fluorophore excreted by cultured phytoplankton (Romera-Castillo et al., 2010).

**Table 2:** Characteristics of fluorescent peaks produced by *A. macleodii*.

Excitation wavelength (nm)	Emission wavelength (nm)	Peak (abbreviation)	Description of previous study
275	300	Tyrosine-like (B)	Tyrosine^1^ Low molecular weight peptide^2^
275	330	Tryptophan-like (T)	Tryptophan^1^ High molecular weight peptide, protein molecule^2^
270	520	Humic-like 1 (H1)	Enriched in humic acid fraction^3,4^
315	400	Humic-like 2 (H2)	Marine autochthonic substance^5^
425	520	Humic-like 3 (H3)	Enriched in humic acid fraction^3,4^

^1^*Mayer et al. (1999); ^2^Yamashita and Tanoue (2004); ^3^He et al. (2006); ^4^Santín et al. (2009); ^5^Coble (1996)*.

### Changes in Fluorescence Intensity of Individual Peaks during *A. macleodii* Incubation

**FIGURE 3:**
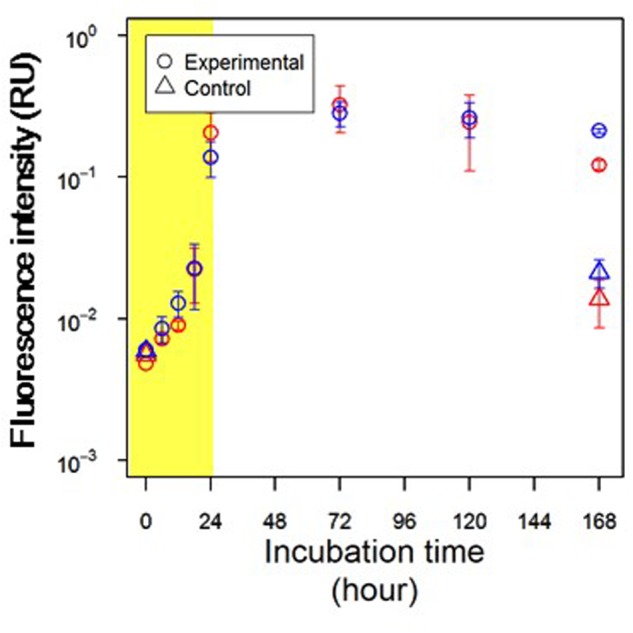
**Changes in fluorescence intensities of protein-like peak**. Red and blue symbols indicate tyrosine-like peak (Ex/Em = 275/300 nm) and tryptophan-like peak (Ex/Em = 275/330 nm), respectively. Circles and triangles represent the experimental and control treatments, respectively. Yellow shading indicates the exponential growth phase.

The fluorescence intensities of two protein-like peaks, namely tyrosine-like and tryptophan-like peaks, increased during the exponential growth phase, particularly during the period of 18–24 h (**Figure 3**), in which the DOC concentration drastically decreased (**Figure 1**). Tyrosine-like fluorescence intensity increased during the early part of the stationary phase (24–72 h, **Figure 3**; Student’s *t*-test, *p* < 0.05) and was tended to decrease during the latter part of the stationary phase (72–168 h; Student’s *t*-test, *p* > 0.05). Tryptophan-like fluorescence intensity decreased significantly during the latter part of the stationary phase (**Figure 3**; Student’s *t*-test, *p* < 0.05).

**FIGURE 4:**
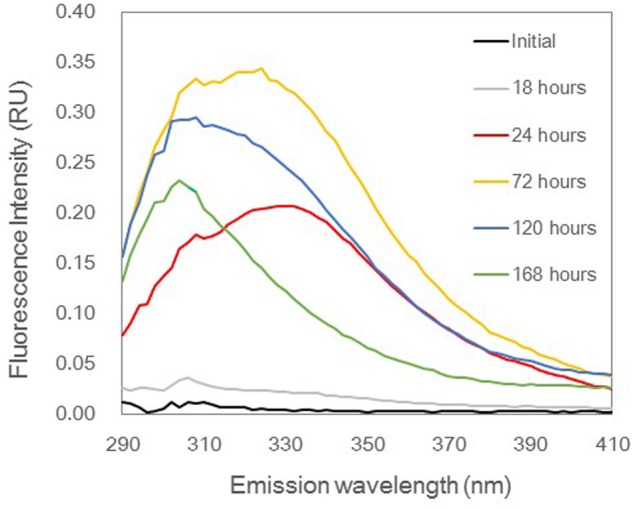
**Emission spectra at 275 nm excitation in the experimental treatment based on the average of triplicate samples**. These spectra indicate changes of protein-like fluorophores. The color of each spectrum represents each incubation time.

Tyrosine-like and tryptophan-like fluorophores exhibited fluorescence peaks with an excitation wavelength of 275 nm (Coble, 2007). It is difficult to quantitatively evaluate changes in protein-like fluorescence intensities, because the emission spectra of tyrosine and tryptophan molecules overlap (Mayer et al., 1999; Lakowicz, 2006). Thus, in this study, the emission spectrum at an excitation wavelength of 275 nm was used to evaluate the dominant protein-like fluorophore during *A. macleodii* incubation (**Figure 4**). The emission spectra were almost the same during 0–18 h, but the fluorescence intensities at wavelengths corresponding to protein-like fluorescence (approximately 290–370 nm) were slightly increased with incubation time (**Figures 3**, **4**). A single fluorophore peaked at 330 nm appeared in 24 h, indicating that tryptophan-like fluorophore was mainly produced during the exponential growth phase (**Figures 2B**, **4**). It seemed that two fluorophores peaked at 330 and 300 nm were the main components of the spectrum at 72 h, indicating that both tryptophan-like and tyrosine-like fluorophores were produced during the early part of the stationary phase (**Figures 2C**, **4**). Although fluorescence intensities corresponding to protein-like fluorophores decreased entirely during 72–168 h, the decrease in the tryptophan-like fluorophore (peak at 330 nm) was greater than that of the tyrosine-like fluorophore (peak at 300 nm) (**Figures 2D**, **4**). Furthermore, the tyrosine-like fluorophore became a major peak at 168 h. The fluorescence intensity at 300 nm should be composed of the peak maximum of tyrosine-like fluorophore and the peak edge of tryptophan-like fluorophore. Thus, changes in the shape of emission spectrum observed during 72–168 h imply that the tryptophan-like fluorophore decreased but the tyrosine-like fluorophore was relatively stable during the latter part of the stationary phase.

**FIGURE 5:**
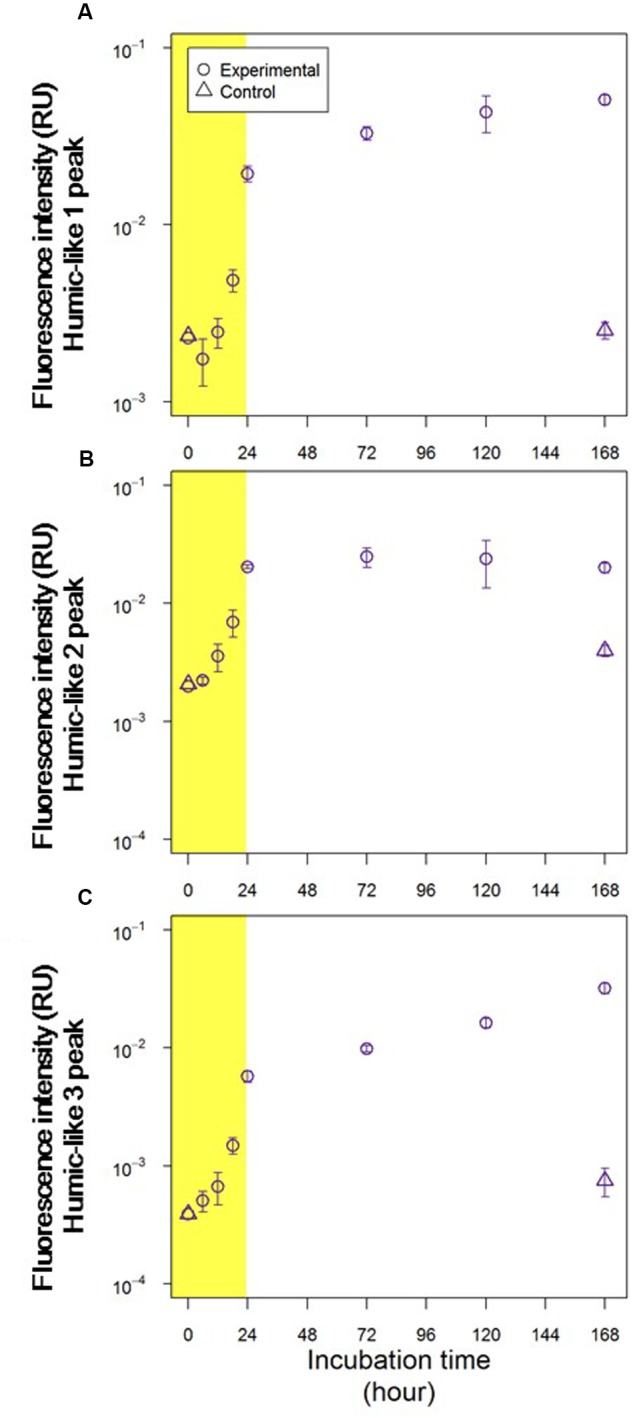
**Changes in fluorescence intensities of (A)** humic-like 1 peak (Ex/Em = 270/520 nm), **(B)** humic-like 2 peak (Ex/Em = 315/400 nm) and **(C)** humic-like 3 peak (Ex/Em = 425/520 nm). Circles and triangles represent the experimental and control treatments, respectively. Yellow shading indicates the exponential growth phase.

The fluorescence intensities of all humic-like fluorophores increased during the exponential growth phase (**Figure 5**). The changes in fluorescence intensity during the stationary phase varied among the humic-like fluorophores (**Figure 5**). Two humic-like fluorophores with emission maxima at longer wavelengths (H1 and H3) continuously increased throughout the stationary phase. In contrast, the other humic-like fluorophore (H2) was relatively stable during the stationary phase (Student’s *t*-test, *p* > 0.05).

Fluorescence intensities of all fluorophores were comparable in the control and experimental treatments at the initiation of these treatments (**Figures 3**, **5**). In the control treatment, the fluorescence intensities of all humic-like fluorophores remained unchanged during the incubation (**Figure 5**). Although the fluorescence intensities of protein-like fluorophores in the control treatment increased several fold during the 168-h incubation, these intensities were 1 order of magnitude lower than those observed for the experimental treatment at the end of the incubation (**Figure 3**).

## Discussion

### Changes in DOM Quantity and Quality with *A. macleodii* Growth

*A. macleodii* has been reported to be a ubiquitous gammaproteobacterium in temperate oceans (López-Pérez et al., 2012). The possibility that *A. macleodii* substantially contributes to the consumption and alteration of labile DOM in surface waters of marine environments has also been considered (Tada et al., 2011; Pedler et al., 2014; Pedler Sherwood et al., 2015). Glucose was used as a labile substrate for *A. macleodii* incubation in this study. Previous studies demonstrated that coastal microbial communities completely consumed glucose within 2 days of incubation (Ogawa et al., 2001; Kawasaki and Benner, 2006). The decrease in the DOC concentration was coupled with the increase in *A. macleodii* abundance, indicating that the decrease in the DOC concentration during the exponential growth phase should be due to the consumption of glucose by the bacterial strain. Assuming that the glucose was completely consumed within several days of incubation, residual DOC was produced by *A. macleodii*.

Protein molecules that contain both tryptophan and tyrosine molecules usually show only tryptophan fluorescence because of energy transfer (Lakowicz, 2006). A previous study analyzed the fluorescence properties of surface DOM in the Sagami Bay and showed that only tryptophan-like fluorescence was evident in high-molecular-weight fractions, whereas tyrosine-like fluorescence was dominant in low-molecular-weight fractions (Yamashita and Tanoue, 2004). Thus, changes in the composition of protein-like fluorophores with *A. macleodii* growth possibly reflected the changes in the relative molecular weight of peptides/proteins, which were released and reused by *A. macleodii*. Tryptophan-like fluorophore clearly appeared at 24 h in our experiment (**Figure 4**), implying that large polymers, such as protein molecules, were predominantly released during the exponential growth phase. The incubations contained not only tryptophan-like fluorophore but also tyrosine-like fluorophore, which both increased during 24–72 h (**Figure 4**). This result suggested that large polymers were still released but that part of these exopolymers were degraded to small peptides. The decrease in tryptophan-like fluorophore was predominant, but tyrosine-like fluorophore was relatively stable at the latter part of the stationary phase (72–168 h), suggesting that the production of small peptides due to the degradation of large exopolymers was correlated with the reutilization of tyrosine-like fluorophores.

The reutilization of exopolymer by *A. macleodii* was suggested in the stationary phase while two humic-like fluorophores continued to accumulate, implying that unavailable DOM were produced from available substrate. The DOC concentrations were relatively stable during the stationary phase, as mentioned above. These results suggested that major portions of DOM produced by *A. macleodii* are unavailable to these bacteria, although a minor fraction of this DOM is reusable by the strain. The remaining DOC concentration produced by *A. macleodii* was estimated to be 41 ± 4 μM C by subtracting the DOC concentration at the end of control treatment (9.6 ± 1.5 μM C; **Figure 1**) from that of experimental treatment (51 ± 4 μM C; **Figure 1**). This remnant DOC concentration corresponded to approximately 4% of the initial glucose used for the *A. macleodii* incubation. Notably, this estimation is comparable to the results obtained by previous studies that conducted incubations of bacterial isolates or microbial communities with glucose. Gruber et al. (2006) showed that 3% of initial glucose was converted to residual DOC by the bacterial isolate *P. chlororaphis* during 36 days of incubation. In addition, 3–6% of initial glucose was found to convert to molecularly uncharacterizable DOM in marine microbial communities, and occurred after 90-day to 2-year periods of incubation (Ogawa et al., 2001; Shimotori et al., 2009; Koch et al., 2014). Such consistency in the production efficiency of residual DOC (i.e., 3–6% of initial substrate) suggests that neither microbial community structure nor microbial growth affected the efficiency of MCP.

### Humic-Like Fluorophores Produced by *A. macleodii*

The production of humic-like fluorophores has been observed in incubation experiments using marine microbial communities (Kramer and Herndl, 2004; Lønborg et al., 2009; Shimotori et al., 2009). This study demonstrated that humic-like fluorophores can be generated not only by microbial communities but also by single bacterial strains using simple substrates, such as glucose. This finding is consistent with those of Shimotori et al. (2012), who found that humic-like fluorophores were generated during incubations of several bacterial isolates in Marine Broth culture medium.

A humic-like fluorophore (H2) produced by *A. macleodii* was similar to a previously defined marine microbial humic-like peak M (Coble, 1996, 2007). This fluorophore also corresponded to a fluorophore that was found to be microbial recalcitrant FDOM with the time scale of the thermohaline circulation (Yamashita and Tanoue, 2008). Fluorescent components similar to H2 were obtained through parallel factor analysis (PARAFAC) of FDOM from the open ocean and were found to be related to apparent oxygen utilization in the deep ocean (Jørgensen et al., 2011; Tanaka et al., 2014), implying that H2 produced by *A. macleodii* is possibly recalcitrant DOM. On the other hand, peak M was produced by phytoplankton isolates (Romera-Castillo et al., 2010) and consumed partially by bacterial communities (Romera-Castillo et al., 2011). These results suggest that the reactivity of the peak M-type fluorophore was dependent on its source; specifically, the fluorophore produced by heterotrophic bacteria is possibly recalcitrant, while that produced by phytoplankton includes a labile fraction.

The other two humic-like fluorophores (H1 and H3), which peaked at longer emission wavelengths than that of H2, continued to be produced by *A. macleodii* during both the stationary phase and the exponential growth phase. Shimotori et al. (2009) also observed a fluorophore peaked at >500 nm emission during the incubation of coastal microbial communities with glucose. On the other hand, there was a high presence of PARAFAC components that were similar to a combination of H1 and H3 in humic acid fractions extracted from soils and sediments (He et al., 2006; Santín et al., 2009). These PARAFAC components were also considered to be terrigenous FDOM (Stedmon and Markager, 2005; Singh et al., 2013). These results suggest that the fluorophore that peaked at a longer emission wavelength (>500 nm) might be ubiquitously produced by heterotrophic bacteria but is enriched in humic acid fractions from soils and sediments. Notably, in Shimotori’s incubation studies (Shimotori et al., 2009), this fluorophore appeared in EEMs during the period of increasing bacterial abundance but disappeared from EEMs during the period of decreasing bacterial abundance. In the open ocean, this fluorophore is generally not present (Jørgensen et al., 2011; Tanaka et al., 2014). Thus, *A. macleodii* may be one of the key species that produces this fluorophore, and this fluorophore might be consumed by other microbes in natural environments.

### Potential Relationship between Bacterial Growth and the Production of Humic-Like Fluorophore

The production rates of microbial humic-like fluorophore differ among substrates used in the incubation of marine bacterial communities (Jørgensen et al., 2014; Aparicio et al., 2015). For example, Aparicio et al. (2015) found that the production rates of marine humic-like fluorophore during the incubation of marine bacterial communities with Suwannee River humic acids were higher than those with glucose and acetate. Using different substrates for microbial community incubation reportedly cause changes in microbial community structure and/or bacterial physiology (Gómez-Consarnau et al., 2012). Therefore, in studies of bacterial communities, it is difficult to evaluate whether the factor regulating the production of humic-like fluorophores is bacterial species or physiology; in contrast, incubations using a bacterial isolate allow insight into the relationship between bacterial physiology and RDOM production.

The bacterial physiology was evaluated in terms of growth phases in this study. The quantity or quality of substrate is one of the critical factors in controlling bacterial growth (Jannasch and Egli, 1993). Therefore, the physiology of *A. macleodii* was likely altered by the change in substrate availability. The exponential growth phase of *A. macleodii* can be characterized as the period that the strain consumed glucose and produced various extracellular compounds, e.g., three humic-like fluorophores as well as a tryptophan-like fluorophore. The physiology of *A. macleodii* shifted to the stationary phase after the complete consumption of glucose, and the tryptophan-like fluorophore was consumed, but two humic-like fluorophores were produced. The results showed that the production mechanisms of humic-like fluorophores, which cannot be reused by the strain, are correlated with the physiology of *A. macleodii*. The humic-like fluorophore (H2) that is similar to recalcitrant microbial humic-like FDOM (Yamashita and Tanoue, 2008; Catalá et al., 2015) is only produced in the exponential phase, but other fluorophores (H1 and H3), which might be consumed by other microbes (Shimotori et al., 2009), are produced during both the exponential and the stationary phases of *A. macleodii*.

The relationship between bacterial growth and RDOM production that is apparent with *A. macleodii* was not consistent with the previous studies conducted by Gruber et al. (2006) and Eichinger et al. (2009). *A. macleodii* was found to produce extracellular humic-like fluorophores, which were unavailable to the strain during the exponential growth and the stationary phases. However, DOC produced by a different *Alteromonas* strain (*A. infernus*) was accumulated under conditions of carbon starvation of the isolate (i.e., the stationary phase in this study) during the incubation in which pyruvate was added every 48-hours as labile substrate (Eichinger et al., 2009). Specific compounds detected by mass spectrometry with electrospray ionization were produced in the exponential growth phase and remained at the end of *P. chlororaphis* incubation with glucose for 36 days, suggesting that recalcitrant compounds, which cannot be reused by the strain, were possibly produced during the exponential growth phase (Gruber et al., 2006). Although incubation conditions, such as temperature, the quality and quantity of substrate, and the composition of artificial seawater, varied among the studies, the results obtained by this study and the previous studies suggest that the physiology related to RDOM production might differ between bacterial species.

## Conclusion and Remarks

The present study first evaluated the relationship between bacterial growth and the production of humic-like fluorophores during the incubation of a model bacterial isolate, *A. macleodii*, with glucose as a substrate. The results of the experiment showed that *A. macleodii* produced three humic-like fluorophores; one is optically similar to recalcitrant microbial humic-like FDOM, while the other two might be consumed by other species of marine bacteria. The compositions of humic-like fluorophores produced by *A. macleodii* differed between the growth phases of the isolate, which might have been affected by the changes in available substrate from glucose to extracellular compounds (such as tryptophan-like fluorophore) released by the isolate.

On the basis of prior studies, variable recalcitrant characteristics are suggested by the humic-like fluorophores produced by *A. macleodii*, although these humic-like fluorophores appeared to be unavailable to the strain. To confirm the recalcitrant characteristics of DOM produced by bacterial isolates (e.g., humic-like fluorophores), the incubation of bacterial DOM with marine microbial communities is necessary. Isolate culture experiments with different incubation conditions (e.g., temperature, substrate quality and quantity) may provide a better understanding of the effect of bacterial physiology on RDOM production. Incubation studies using several species of bacterial isolates with the same conditions are also necessary to confirm whether the relationship between bacterial physiology and RDOM production differs among bacterial species. The cumulative results of such in vitro experiments will provide better insight into the relationship between bacterial physiology and RDOM production, and thus, such experiments will contribute to a better understanding of the factors that regulate the efficiency of MCP, which potentially shapes the pool size and composition of marine DOM.

## Author Contributions

All authors contributed to the design of the study. SG performed incubation experiment, sample measurements, and data analyses with help of YT, KS, and YY. SG wrote the initial draft of the manuscript and all authors contributed to its revision.

## Conflict of Interest Statement

The authors declare that the research was conducted in the absence of any commercial or financial relationships that could be construed as a potential conflict of interest.
